# Activity of Lipase and Chitinase Immobilized on Superparamagnetic Particles in a Rotational Magnetic Field

**DOI:** 10.1371/journal.pone.0066528

**Published:** 2013-06-14

**Authors:** Toru Mizuki, Miyuki Sawai, Yutaka Nagaoka, Hisao Morimoto, Toru Maekawa

**Affiliations:** Bio-Nano Electronics Research Centre, Toyo University, Saitama, Japan; Brandeis University, United States of America

## Abstract

We immobilize hydrolases such as lipase and chitinase on superparamagnetic particles, which are subjected to a rotational magnetic field, and measure the activities of the enzymes. We find that the activities of lipase and chitinase increase in the rotational magnetic field compared to those in the absence of a magnetic field and reach maximum at certain frequencies. The present methodology may well be utilized for the design and development of efficient micro reactors and micro total analysis systems (μ-TASs).

## Introduction

The interactions between enzymes and nano/micro particles have been intensively studied in recent years in order to immobilize enzymes on particles [1]–[6]. It is now well known that the activities of those enzymes can be, in general, stabilized under a wide range of environmental conditions such as the pH and temperature although the actual activities of enzymes are usually reduced after having been immobilized on particles [7]–[12]. Magnetic particles are often used in bio-medical experiments for the labeling, manipulation or sorting of biomolecules and cells [13]–[15]. It was recently demonstrated that the activity of α-amylase immobilized on superparamagnetic particles was increased in a rotational magnetic field and that the activity reached maximum at a certain frequency [16]. An interesting question arises; that is, do the above features apply universally to the other hydrolases or only to α-amylase as a specific case? The structures of clusters composed of magnetic particles are determined by the control parameter 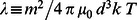, where *m*, *μ*_0_, *d*, *k* and *T* are, respectively, the absolute value of the magnetic dipole moment of a particle, the magnetic permeability of a solvent, the diameter of a particle, the Boltzmann constant and the temperature [17]–[20]. *λ* represents the ratio of magnetic dipole-dipole interactive energy to thermal energy. It is known that clusters are formed by magnetic particles when *λ* is greater than 5 even in the absence of a magnetic field and the growth rate and structures of the clusters change depending on *λ* and the volume fraction of the magnetic particles *φ* [17]. The control parameter, which represents the ratio of viscous drag to magnetic force acting on a cluster in a rotational magnetic field, is the Mason number, which is defined by 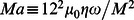, where *η*, *ω* and *M* are, respectively, the dynamic viscosity of a solvent, the angular frequency of a rotational magnetic field and the magnetization of a particle. Chain clusters tend to be broken due to viscous drag as the Mason number increases [18]–[21]. In this article, we immobilize lipase and chitinase; enzymes capable of hydrolyzing lipid and chitin [22], [23], on superparamagnetic particles and investigate the effect of the frequency of a rotational magnetic field on the activities of the enzymes. We discuss the basic idea and recipe for the control of the activity of the hydrolases via immobilization on superparamagnetic particles and application of an external rotational magnetic field.

## Materials and Methods

We immobilized lipase A from *Candida antarctica* (Wako Pure Chemical Industries Ltd.) on superparamagnetic particles (Nanomag-D Plain, Micromod Partikeltechnologie GmbH) by mixing the particles with lipase dispersed solution at 4°C for 10 h. We also immobilized chitinase from *Trichoderma viride* (Sigma-Aldrich Inc.) on superparamagnetic particles by mixing the particles with chitinase dispersed solution at 4°C for 10 h. For the immobilization of lipase and chitinase on superparamagnetic particles, the volume fraction of the particles in the solution *φ* was set at 6.2×10^−3^ and the mass concentrations of lipase and chitinase in the solution were, respectively, set at 0.5 and 5.0 mg ml^−1^. The average diameter and magnetic dipole moment of each particle were 130 nm and 3.3×10^−23^ Wb m. The surface of the particle was not modified with any molecules such as amino or carboxyl bases. The enzyme/particle hybrids were collected by a magnet and washed three times with water. The number of lipase molecules remaining in the solution was estimated by measuring the absorbance of 280 nm photons, from which we calculated the average number of lipase molecules immobilized on each superparamagnetic particle, which was 370. The number of chitinase molecules immobilized on each particle was also calculated in the same way as in the case of lipase molecules and it was 2070.

**Figure 1:**
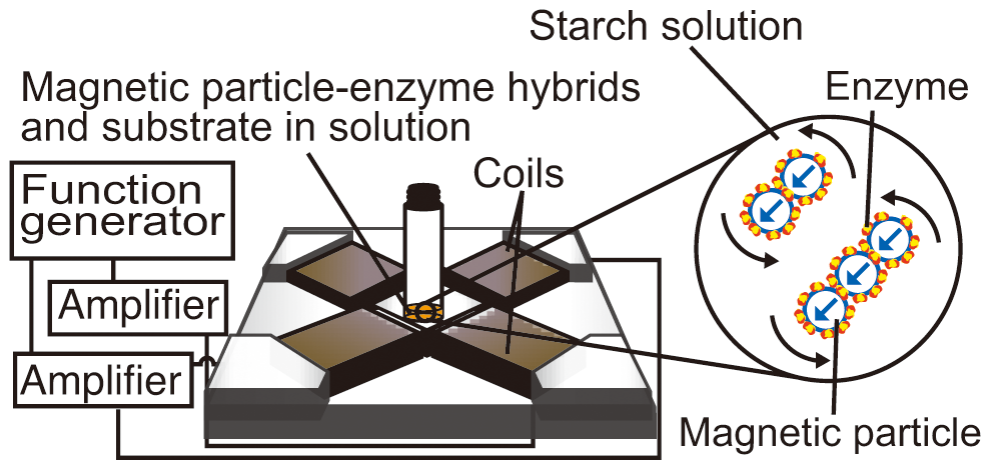
Outline of the experimental system. Lipase and chitinase molecules are immobilized on superparamagnetic particles, the average diameter and magnetic dipole moment of which are 130 nm and 3.31×10^−23^ Wb m, by mixing the particles with aqueous solution, in which lipase and chitinase molecules are dispersed. Dc and rotational magnetic fields are generated by modulating the phase of the electric current supplied to each pair of electromagnets using a function generator. The strength of the magnetic field is set at 9.55 kA m^−1^, which corresponds to a magnetic flux density of 12 mT, and the frequency of the magnetic field is changed; 1, 3, 5, 7, 10 and 30 Hz. The enzyme-substrate reaction experiment is carried out at 25°C for 30 minutes and the enzyme activity is estimated by measuring the absorbance of 410 nm photons.

A rotational magnetic field was produced by supplying an electric current to two orthogonal pairs of electromagnets using two power amplifiers and a function generator (see Figure 1). The details of the generation of the rotational magnetic field are summarized in Refs. [18], [19]. We introduced the superparamagnetic particles, on which lipase or chitinase molecules had been immobilized, into test tubes, which were placed in a test area surrounded by two orthogonal pairs of electromagnets. The volume fraction of the particles *φ* was set at 7.0×10^−4^ in both lipase and chitinase cases. For the measurement of the activity of lipase, 15 µl of solution, in which lipase/particle hybrids were dispersed, was mixed with 360 µl of substrate solution, which was composed of 0.04% *p*-nitrophenyl palmitate, 2% TritonX and 50 mM Tris-HCl buffer of pH 7.6, while in the case of chitinase, 50 µl of solution, in which chitinase/particle hybrids were dispersed, was mixed with 300 µl of substrate solution composed of 1 mM *p*-nitrophenyl N-acetyl-β-D-glucosaminde acid and 100 mM Tris-HCl buffer of pH 7.6. The test tubes were set in a rotational magnetic field of 1, 3, 5, 7, 10 and 30 Hz at 25°C for 30 minutes. The strength of the magnetic field was 9.55 kA m^−1^, which corresponds to a magnetic flux density of 12 mT. The quantity of p-nitrophenol, which had been released after the enzyme/substrate reactions, was estimated by measuring the absorbance of 410 nm photons, from which the activities of lipase and chitinase were calculated. A kinetic constant *k_cat_*; i.e., the number of catalytic turnover events occurring per unit time, was also estimated [24] and the dependence of the activities and *k_cat_* on the frequency of the rotational magnetic field was clarified. We measured the zeta potential and the distributions of the hydrodynamic diameters of the enzyme/particle hybrids in the solution by the laser dynamic scattering method (Zetasizer nano-zs, Malvern Instruments Ltd.) and observed the dynamics and structures of the clusters formed by the enzyme/particle hybrids by a high-speed CCD camera (VW-5000, Keyence Co.) mounted on an inverted microscope (TE-2000-U, Nikon Co.).

## Results and Discussion

As we mentioned, enzymes such as lipase and chitinase were immobilized on the surface of superparamagnetic particles and the effect of the immobilization on the activities of the enzymes was investigated in the absence or presence of a rotational magnetic field. First of all, we confirmed that there was no appreciable effect of dc or rotational magnetic fields on the activities of lipase and chitinase, which were not immobilized on superparamagnetic particles, since the magnetic field was as weak as 9.55 kA m^−1^, which corresponds to a magnetic flux density of 12 mT. Note that the activities of enzymes can be raised by applying an extremely strong uniform or gradient magnetic field to the enzyme dispersed solutions [25]. The activities of lipase and chitinase immobilized on the superparamagnetic particles were reduced, respectively, by approximately 30 and 90% in the absence of a magnetic field compared to those of lipase and chitinase without immobilization on the particles as in most of the cases [7]–[12]. Note that the activity of α-amylase immobilized on the same superparamagnetic particles was the same as that without immobilization on the particles in the absence of a magnetic field [16].

**Figure 2:**
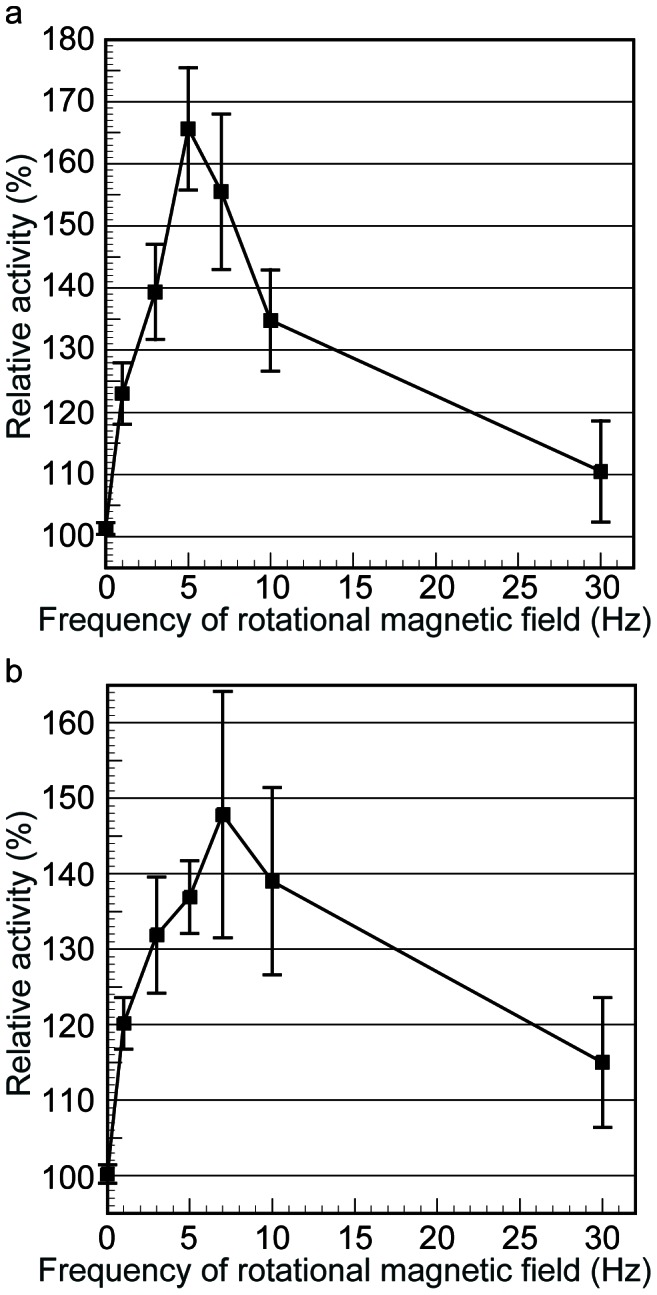
Dependence of the relative activities on the frequency of the external rotational magnetic field. (a) Lipase. (b) Chitinase. The ordinate axis represents the activity of the enzymes immobilized on particles in a rotational magnetic field, which is normalized by that in the absence of a magnetic field. The diameter of each particle is 130 nm. The strength of the magnetic field is 9.55 kA m^−1^. The activities increase and become maximum at certain frequencies.

The dependence of the activities of lipase and chitinase on the frequency of the rotational magnetic field is shown in Figure 2, where the ordinate axis represents the activity of those enzymes immobilized on superparamagnetic particles in a rotational magnetic field, which was normalized by that in the absence of a magnetic field. The activities of lipase and chitinase increased in the rotational magnetic field compared to those in the absence of a magnetic field. In the case of lipase, the activity became maximum at a frequency of 5 Hz, whereas in the case of chitinase, it reached maximum at 7 Hz.

We measured the zeta potentials and size distributions of the enzyme/particle hybrids in the solution by the laser dynamic scattering method as mentioned and confirmed that those hybrids were, statistically speaking, monodispersed stably in the solution in the absence of a magnetic field due to the low volume fraction of the particles. The average zeta potential and hydrodynamic diameter of the lipase/particle hybrid were, respectively, −1.65 mV and 166 nm, whereas those of the chitinase/particle hybrid were −17.1 mV and 173 nm. Note that the average zeta potential and hydrodynamic diameter of the bare particle were −4.0 mV and 130 nm. We suppose that lipase and chitinase were successfully immobilized on the particles via Coulomb interactions, noting that the particles are negatively charged and some domains in the enzymes are positively charged (Appendix S1). Snapshots of the clusters formed by lipase/particle and chitinase/particle hybrids in the rotational magnetic field are shown in Appendix S2. Rod clusters were formed via chain-chain coagulations as in the case of α-amylase [16]. As the frequency increased up to the one corresponding to the maximum activity, the length of the clusters decreased with an increase in the frequency of the rotational magnetic field and the clusters rotated without any delay following the external rotational magnetic field, in which case the enzymes' activity increased with an increase in the frequency of the rotational magnetic field. However, those clusters were broken into much smaller ones once the frequency of the rotational magnetic field exceeded the one corresponding to the maximum activity. Note that a single superparamagnetic particle does not rotate around its center of inertia in a uniform rotational magnetic field and therefore it does not contribute to any increase in the enzyme activity. The value of the control parameter *λ* was 7.7 in the present case, which was obviously large enough for those clusters to be formed [17]–[21]. The values of the Mason number, at which the activities reached maximum, were, respectively, 6.1×10^−3^ (5 Hz) and 8.6×10^−3^ (7 Hz) in the cases of lipase and chitinase. The clusters were broken even when the Mason number was as low as 6.1×10^−3^ and 8.6×10^−3^ since the local volume fraction in the clusters was high [18], [21].

There are two important factors for the increase in the enzyme activity; that is, (a) the formation of large clusters composed of enzyme/particle hybrids, in which case a large number of enzyme molecules can participate in the reaction, and (b) a higher rotational frequency of the clusters, in which case the collision rate of enzyme molecules with substrate increases. Since the clusters are broken into smaller ones or individual single particles due to viscous drag acting on the clusters as the frequency increases as mentioned above, there is an optimal frequency for the increase in the activity. The optimal frequencies were, respectively, 5 and 7 Hz in the cases of lipase and chitinase in the present study. We suppose that in the case of lipase, the clusters were dissociated at lower frequency than in the case of chitinase since surfactant; i.e., TritonX, was mixed with the lipase solution for dissolving the substrate molecules. We checked the effect of both rotational stirring and translational shaking of the solutions on the activities of the above enzymes using a conventional stirrer and shaker, but no appreciable increase in the activities was detected since the enzymes and substrate molecules rotate or oscillate at the same phase in the solution, in which case the collision rate of enzymes with substrate molecules is the same as that in the stationary case. We suppose that relative motion between enzymes and substrate molecules is necessary for the increase in the activity. In the present case, enzymes immobilized on particles were rotated in the stationary solution, in which substrate molecules were dissolved, in which case the collision rate of the enzymes with substrate molecules increases. The value of the kinetic constant *k_cat_*, in fact, increased with an increase in the frequency of the rotational magnetic field up to, respectively, 5 and 7 Hz in the cases of lipase and chitinase; i.e., *k_cat_* (lipase) = 3.23 min^−1^ at 0 Hz, 9.60 min^−1^ at 3 Hz and 1.39×10 min^−1^ at 5 Hz, and *k_cat_* (chitinase) = 8.58×10 min^−1^ at 0 Hz, 1.28×10^2^ min^−1^ at 5 Hz and 1.30×10^2^ min^−1^ at 7 Hz.

It is known that the activities of enzymes are, in general, reduced but the stability is improved once enzymes are immobilized on particles [1]–[12]. Several innovative enzyme immobilization methodologies for not lowering the activities have been proposed [1]–[6]. According to the present and previous studies [16], enzymes can be immobilized on superparamagnetic particles via electrostatic interactions, noting that the surface of the particles was not modified with any molecules, and the activities of lipase and chitinase were reduced, whereas the activity of α-amylase was not decreased in the absence of a magnetic field even after immobilization. The activities of the enzymes may be affected by the positions of the active sites in the enzymes immobilized on the surface of the particles. When the active sites are open directing towards the substrate molecules dispersed in the solution, the activity of the enzymes would be high and vice versa. We will be calculating the charge distributions on the surface of enzymes in detail so that the configurations of the enzymes immobilized on particles can be precisely estimated. We found that an application of a rotational magnetic field to the solution, in which enzyme/particle hybrids and substrate molecules are dispersed, raises the activities of α-amylase, lipase and chitinase compared to those in the absence of a rotational magnetic field. The optimal frequencies corresponding to the maximum activity may be changed depending on the control parameters such as *φ*, *λ* and *Ma*. Another advantage of the present method is that enzyme/particle hybrids can be collected by applying a gradient magnetic field so that the enzymes can be reused. We will be systematically investigating the effect of the above parameters; i.e., *φ*, *λ* and *Ma*, on the activities of enzymes and the effect of other types of magnetic nanomaterials such as magnetic carbon nanotubes [19], [26], [27] and magnetic carbon onions [28],[29] on the efficiency of immobilization and the increase in the activity. We will also be investigating the effect of immobilization and rotational magnetic fields on the activities of the other types of enzymes such as oxidoreductases, transferases, lyases, isomerases, ligases and so on. The present methodology may well be utilized for the design and development of efficient micro reactors and micro total analysis systems (μ-TASs).

## Supporting Information

Appendix S1 **Charge distributions on the surface of enzymes.**
(DOCX)

Appendix S2 **Clusters formed by enzyme/superparamagnetic particle hybrids.**
(DOCX)
