# Supplementary material for: Activity of Lipase and Chitinase Immobilized on Superparamagnetic Particles in a Rotational Magnetic Field
Source: PLoS One. 2013 Jun 14;8(6):e66528. doi: 10.1371/journal.pone.0066528 (PMC3682989; doi:10.1371/journal.pone.0066528)
Supplement: Appendix S1 — Charge distributions on the surface of enzymes. (DOCX) [file pone.0066528.s001.docx]

## Online Supplementary Material

**Appendix S1: Charge distributions on the surface of enzymes**

Activity of lipase and chitinase immobilized on superparamagnetic particles in a rotational magnetic field

Toru Mizuki, Miyuki Sawai, Yutaka Nagaoka, Hisao Morimoto and Toru Maekawa^†^

Bio-Nano Electronics Research Centre, Toyo University, Saitama 350-8585, Japan.

^†^ E-mail: maekawa@toyo.jp

We calculated the charge distributions on the surface of lipase, chitinase and α-amylase dispersed in water of pH=7 using ®DiscoveryStudio (Ver3.5, Accelrys Inc.). Figure S1 shows the structures of lipase, chitinase and α-amylase, where the active sites are indicated in yellow except (b), and the charge distributions on the surface of the enzymes, where negatively charged domains are shown in red and positively charged ones in blue.


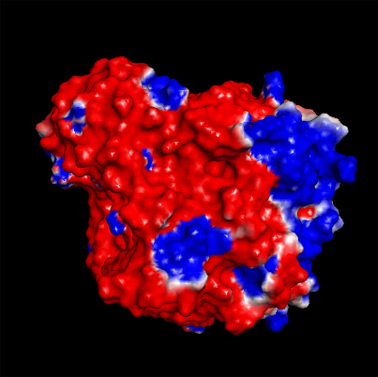

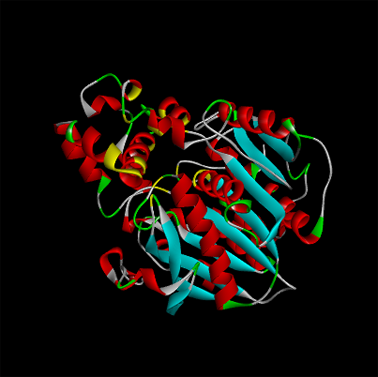


Figure S1 Structures of enzymes (left images) and charge distributions on the surface of the enzymes (right images). (a) Lipase A from *Candida antarctica* (PDB ID: 3GUU); (b) Homology modeling of chitinase from *Trichoderma viride* (Accession No. AF208842); (c) *α*-amylase from *Bacillus licheniformis* (PDB ID: 1BLI). The active sites are indicated in yellow except (b) (left images). Negatively charged domains are shown in red, whereas positively charged ones in blue (right images).

a


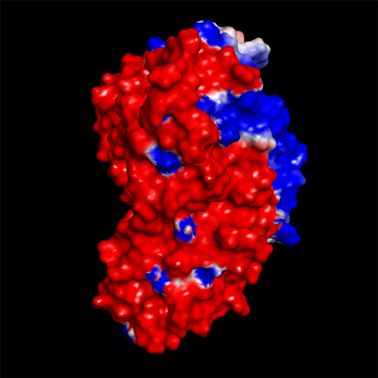

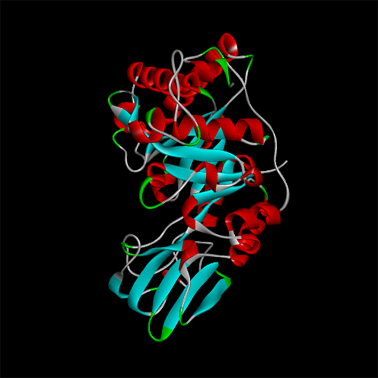


b


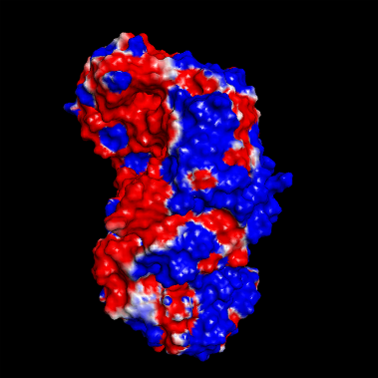


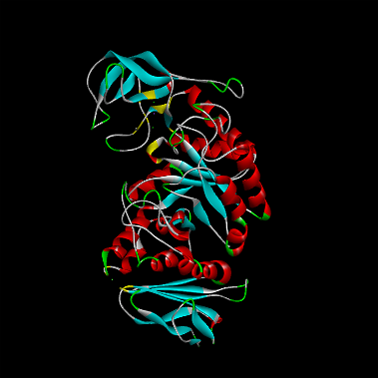


cb
