# Supplementary material for: Activity of Lipase and Chitinase Immobilized on Superparamagnetic Particles in a Rotational Magnetic Field
Source: PLoS One. 2013 Jun 14;8(6):e66528. doi: 10.1371/journal.pone.0066528 (PMC3682989; doi:10.1371/journal.pone.0066528)
Supplement: Appendix S2 — Clusters formed by enzyme/superparamagnetic particle hybrids. (DOCX) [file pone.0066528.s002.docx]

## Online Supplementary Material

**Appendix S2: Clusters formed by enzyme/superparamagnetic particle hybrids**

Activity of lipase and chitinase immobilized on superparamagnetic particles in a rotational magnetic field

Toru Mizuki, Miyuki Sawai, Yutaka Nagaoka, Hisao Morimoto and Toru Maekawa^†^

Bio-Nano Electronics Research Centre, Toyo University, Saitama 350-8585, Japan.

^†^ E-mail: maekawa@toyo.jp

We observed the dynamics and structures of the clusters formed by the enzyme/particle hybrids by a high-speed CCD camera mounted on an inverted microscope. Snapshots of the clusters formed by enzyme/superparamagnetic particle hybrids are shown in Figure S2. Rod clusters were formed via chain-chain coagulations and rotated following the rotational magnetic field without any delay. The length of the clusters decreased with an increase in the frequency of the magnetic field. The clusters were broken into much smaller ones when the frequencies of the magnetic field exceeded the ones corresponding to the maximum activities.


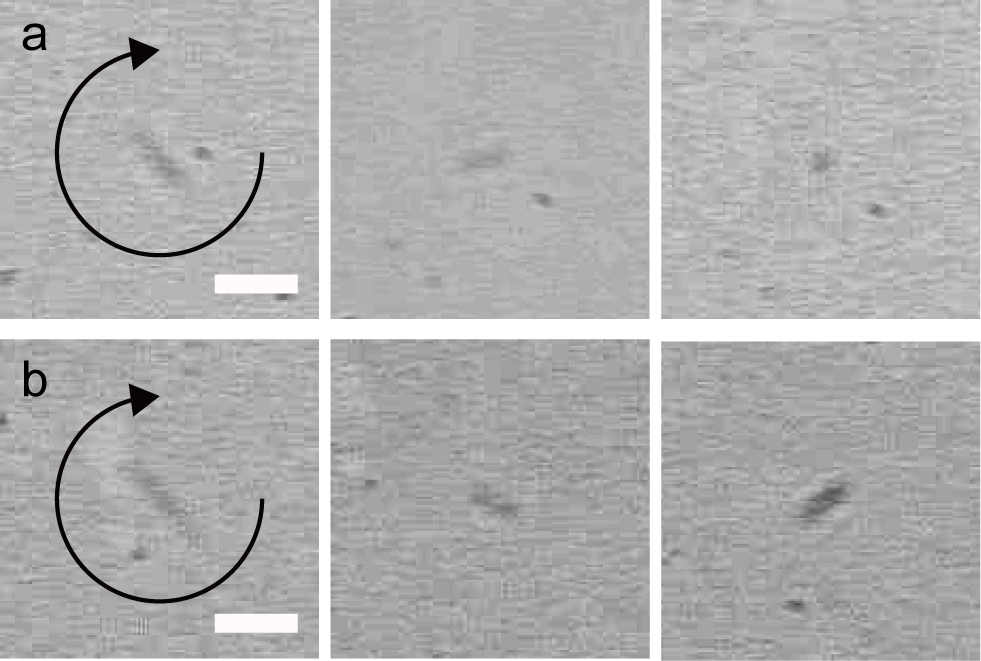


Figure S2 Clusters formed by enzyme/superparamagnetic particle hybrids. (a) Cluster formed by lipase/superparamagnetic particle hybrids. (b) Cluster formed by chitinase/superparamagnetic particle hybrids. The frequency of the rotational magnetic field is 1 Hz (left photos), 3 Hz (middle) and 5 Hz (right). The scale bars represent 2 µm.
